# Differential Activities of the Botanical Extract PBI-05204 and Oleandrin on Innate Immune Functions under Viral Challenge Versus Inflammatory Culture Conditions

**DOI:** 10.3390/molecules28124799

**Published:** 2023-06-16

**Authors:** Gitte S. Jensen, Liu Yu, Ifeanyi Iloba, Dina Cruickshank, Jose R. Matos, Robert A. Newman

**Affiliations:** 1 NIS Labs, 1437 Esplanade, Klamath Falls, OR 97601, USA; ifeanyi@nislabs.com; 2 NIS Labs, 807 St. George St., Port Dover, ON N0A 1N0, Canada; yuliuliu1995@outlook.com (L.Y.); dina@nislabs.com (D.C.); 3 Phoenix Biotechnology, 8626 Tesoro Drive, Suite 801, San Antonio, TX 78217, USA; patents@innovarllc.com (J.R.M.); newmanscientificconsulting@gmail.com (R.A.N.)

**Keywords:** anti-inflammatory, CD25, CD69, CD107a, immune-modulation, interferon-gamma, NK cells, cytotoxic T cells, oleandrin, *Nerium oleander*

## Abstract

The *Nerium oleander* extract PBI 05204 (PBI) and its cardiac glycoside constituent oleandrin have direct anti-viral properties. Their effect on the immune system, however, is largely unknown. We used an in vitro model of human peripheral blood mononuclear cells to document effects under three different culture conditions: normal, challenged with the viral mimetic polyinosinic:polycytidylic acid Poly I:C, and inflamed by lipopolysaccharide (LPS). Cells were evaluated for immune activation marks CD69, CD25, and CD107a, and culture supernatants were tested for cytokines. Both PBI and oleandrin directly activated Natural Killer (NK) cells and monocytes and triggered increased production of cytokines. Under viral mimetic challenge, PBI and oleandrin enhanced the Poly I:C-mediated immune activation of monocytes and NK cells and enhanced production of IFN-γ. Under inflammatory conditions, many cytokines were controlled at similar levels as in cultures treated with PBI and oleandrin without inflammation. PBI triggered higher levels of some cytokines than oleandrin. Both products increased T cell cytotoxic attack on malignant target cells, strongest by PBI. The results show that PBI and oleandrin directly activate innate immune cells, enhance anti-viral immune responses through NK cell activation and IFN-γ levels, and modulate immune responses under inflamed conditions. The potential clinical impact of these activities is discussed.

## 1. Introduction

The continuous emergence of pathogenic viruses and the increased rate of re-emergence of new variants has become a major global threat not only to human health but to commercial animal production as well [1,2,3]. Present therapies targeting viral infections mainly rely on either an adaptive immune response (involving the formation of antibodies against specific viral antigens) or a direct drug-related antiviral activity [4,5]. While immune targeted responses relying on vaccines, for example, have proven to be beneficial, their relative effectiveness largely depends on the functional state of an individual’s immune system which tends to be impaired during senescence and infancy or even compromised in certain health conditions [6,7]. Moreover, because the body’s response to a viral infection can overwhelm the immune system, effective control of viral infections requires the appropriate level of activation of key components of the immune system, particularly the innate immune system [8,9].

The innate immune system, however, has been described as a double-edged sword. On the one hand, it is vital for inducing an effective early control of invading viruses. On the other hand, it has been shown to often result in hyper-inflammatory responses and events characterized as cytokine storms which may lead to adverse health issues. Given the cells and molecules involved, innate immunity is clearly a silent, unperceived, yet necessary physiological inflammatory response. However, if the host response is excessive, it may lead to uncontrolled and surplus release of pro-inflammatory molecules that can be considered as pathological [10,11]. This damage may result further in the loss of function of an organ such as the lung due to the deleterious inflammatory reaction induced by the host immune response rather than the direct effect of an infection. Consequently, a balanced pro-inflammatory/anti-inflammatory approach to the treatment of viral infections would be clinically important. It would ensure that the induction of a strong innate immune response is concomitantly controlled with an anti-inflammatory effect such that excessive inflammation is reduced and potential damage to target organs and tissues is minimized or even prevented. The potential benefits of such a balanced approach have led to the development of a rationale for using certain anti-inflammatory phytochemicals against virus-induced hyperinflammatory responses [12,13].

While the development of vaccines against targeted viruses will continue to play a critical role in combating viral-mediated disease, they are limited because of the constant requirement for specificity to target viruses, in addition to the high cost of developing new vaccine variants against viral mutants. Moreover, vaccines are targeted to specific viral variants and rarely afford broad protection against a viral species. Fortunately, the broader antiviral potential of selected plants and their isolated natural compounds is now appreciated [13,14,15]. Because natural products can be isolated rather than synthesized, they are generally a more effective remedy against viral infections as demonstrated by their mode of action which is fundamentally different from the single viral drug-target pharmaceutical approach. This is primarily due to the synergistic antiviral activities of the multiple phytochemicals contained in natural products, which can potentially target different viral variants simultaneously, a feat that can contribute significantly to reducing the cost of treatment. Recent reviews of the antiviral effects of compounds such as those that cause Na^+^/K^+^-ATPase inhibition discussed the actions of various cardiac glycosides such as oleandrin against a wide variety of viruses including cytomegalovirus, herpes simplex virus I and II, adenovirus, Chikungunya virus, coronaviruses (SARS-CoV-2), respiratory syncytial virus, Ebola virus, Marburg virus, Influenza virus, the human T-cell leukemia virus type-1 (HTLV-1), and the human immunodeficiency virus type-1 (HIV-1) [16]. 

Beyond their well-recognized pharmacologic use as agents that affect the cardiovascular system, the role of cardiac glycosides as anti-inflammatory agents has only recently received attention. Furst et al. described the effects of agents, such as digoxin’s ability to inhibit inflammation in both in vivo and in vitro models [13]. They reported the ability of cardiac glycosides to selectively inhibit acute and infection-induced inflammation, chronic inflammatory autoimmune diseases, and neuroinflammation. Additional anti-inflammatory effects of cardiac glycosides have been reported, e.g., inhibition of fluid extravasation, leukocyte infiltration, TH17 cell differentiation, cytokine secretion, nuclear transcription factor kappa β (NF-κβ) activation, and inhibition of nuclear receptor RORγt [17,18]. A major contributor to adverse inflammatory responses is the transcription factor NF-κβ. We and others have shown that oleandrin suppresses activation of NF-κβ, activator protein-1, and c-Jun NH2 terminal kinase, key regulators of inflammation and cancer, thus providing evidence of major anti-inflammatory mechanisms of action for this compound [19].

While oleandrin has been shown to impact an adaptive immune response against human malignant cells through induction of immunogenic cell death [20,21], the role of oleandrin in inducing an enhanced innate immune response is unclear. Our previous research demonstrated that topical application of herbal extracts containing oleandrin resulted in beneficial antioxidant protection in several cellular models without the induction of leukocyte activation and secretion of inflammatory cytokines [22]. The present research sought to understand the effects of oleandrin and a botanical extract of *Nerium oleander* containing this molecule on blood-derived immune cells obtained from healthy human volunteers. In addition, the effects on innate immune cells as well as on inflammatory responses were examined when cells were exposed to either viral or inflammatory mimetic challenges. The experimental model involved primary immune cells from peripheral blood of healthy donors, where the cell cultures allow cross-talk between monocytes/macrophages, natural killer (NK) cells, and T lymphocytes, to mimic the cross-talk that happens in lymphoid and mucosal tissues, following the methodology from previous publications from our team [23,24,25,26]. 

Since, the mucosal innate immune system acts as both a physical and immunological barrier, which plays a key role in sensing and eliminating pathogens as a first line of defense against inhaled or ingested pathogens, the introduction of immunogenic natural compounds onto mucosal surfaces offers a promising non-invasive method of activating the innate immune cells in the mucosa. The data presented herein demonstrate the ability of oleandrin, at nontoxic very low concentrations, i.e., nanomolar concentrations, to induce a potent functional activation of the innate immune system combined with a complementary anti-inflammatory activity. 

## 2. Results

### 2.1. Induction of the CD69 and CD25 Activation Markers on Innate Immune Cells

**Figure 1:**
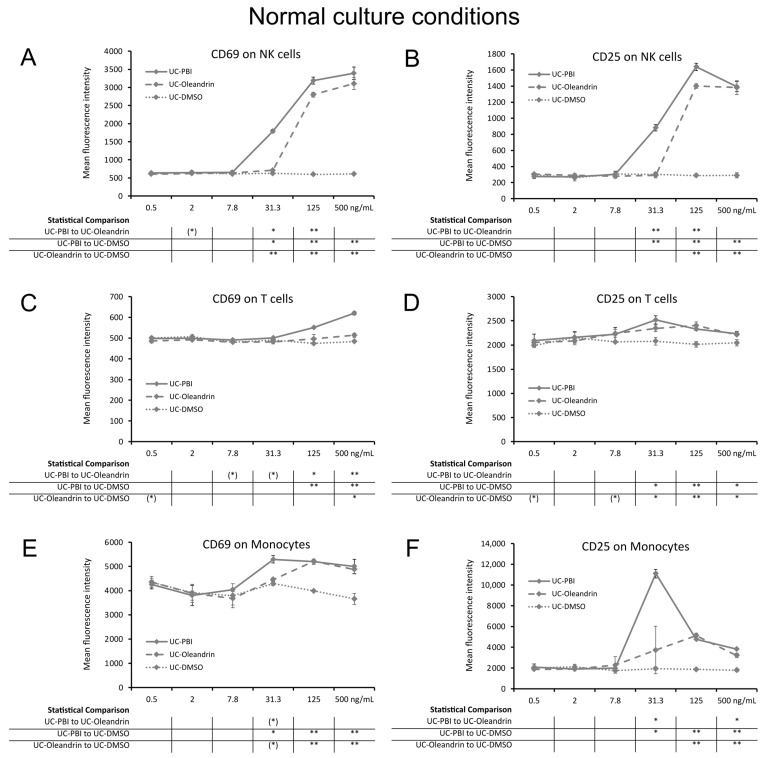
Expression of the activation markers CD25 and CD69 on human immune cell subsets from 24 h PBMC cultures under normal culture conditions, where cells were treated with PBI (solid lines), oleandrin (dashed lines), and untreated control cultures treated with matching concentrations of DMSO (UC-DMSO) (dotted lines). (**A**) CD69 expression on natural killer (NK) cells. (**B**) CD25 expression on NK cells. (**C**) CD69 expression on T cells. (**D**) CD25 expression on T cells. (**E**) CD69 expression on monocytes. (**F**) CD25 expression on monocytes. Since both PBI and oleandrin were solubilized in DMSO, a matching concentration range of DMSO served as a solvent control. The results shown are representative of six repeats from different blood donors. For each concentration of a test product, results are shown as the average ± standard deviation of triplicate samples. In the table below the graph, statistical significance at different concentrations is indicated by asterisks, when *p* < 0.10: (*), *p* < 0.05: *, *p* < 0.01: **.

The botanical extract from *Nerium oleander*, PBI 05204 (PBI) and the pure compound oleandrin showed similar innate immune cell activating properties (Figure 1). The results indicate that the peripheral blood mononuclear cells (PBMC) treated with both test products triggered increased expression of the CD69 and CD25 activation markers on NK cells (Figure 1A,B), T cells (Figure 1C,D), and monocytes (Figure 1E,F). The activating effect was most pronounced for NK cells and monocytes and mild for T cells. The increased expression of CD69 on NK cells treated with PBI was highly significant at the three highest concentration, whereas for oleandrin this was seen for the two highest concentrations (*p* < 0.01).

For CD69 expression and CD25 expression, a broader concentration range was detected for PBI for NK cell activation than for pure oleandrin. The increase in CD69 and CD25 expression for PBI-induced NK cell activation (Figure 1A,B) was detected at >7.8 ng/mL PBI, whereas that of pure oleandrin was detected at a concentration of >31.3 ng/mL, suggesting that the immune-activating effects of PBI are due to its content of oleandrin and that the additional immune-activating effects may be due to one or more other compounds in the extract.

In addition, effects of PBI on CD25 expression on T cells (Figure 1D) and of both CD69 and CD25 on monocytes (Figure 1E,F) were detected at a concentration of 31.3 ng/mL. The increased expressions of CD69 and CD25 on monocytes (Figure 1E,F) were highly significant at higher concentrations >31.3 ng/mL for both PBI and oleandrin (*p* < 0.01).

### 2.2. Enhanced Expression of CD69 and CD25 Activation Markers with Viral Mimetic Challenge

**Figure 2:**
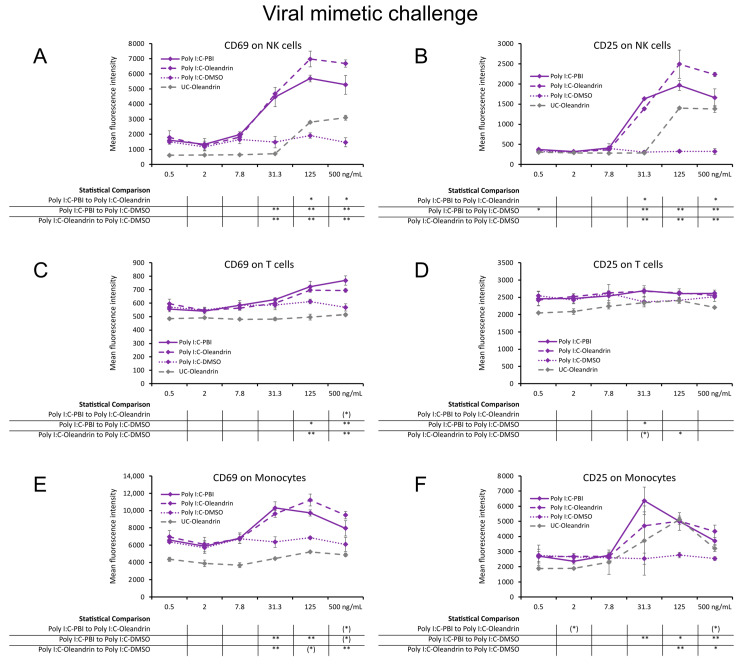
Expression of the activation markers CD25 and CD69 on human immune cell subsets from 24 h PBMC cultures under a viral mimetic challenge, where the cells were treated with Poly I:C-PBI (solid lines), Poly I:C-oleandrin (dashed lines), and Poly I:C control cultures treated with matching concentrations of DMSO (Poly I:C-DMSO) (dotted lines), followed by a viral mimetic challenge by polyinosinic:polycytidylic acid (Poly I:C). (**A**) CD69 expression on natural killer (NK) cells. (**B**) CD25 expression on NK cells. (**C**) CD69 expression on T cells. (**D**) CD25 expression on T cells. (**E**) CD69 expression on monocytes. (**F**) CD25 expression on monocytes. Since both PBI and oleandrin were solubilized in DMSO, a matching concentration range of DMSO served as a solvent control (Poly I:C-DMSO), reflecting the levels of CD69 and CD25 in cultures treated with Poly I:C and the matching concentrations of DMSO. The results shown are representative of six repeats from different blood donors. For each concentration of a test product, results are shown as the average ± standard deviation of triplicate samples. In the table below the graph, statistical significance at different concentrations is indicated by asterisks, when *p* < 0.10: (*), *p* < 0.05: *, *p* < 0.01: **.

In PBMC cultures challenged with viral mimetic polyinosinic:polycytidylic acid (Poly I:C), cells that were pre-treated with PBI and pure oleandrin before the Poly I:C challenge showed stronger immune activating properties than Poly I:C alone (Figure 2). The expression levels of CD69 and CD25 on Poly I:C challenged NK cells (Figure 2A,B) were strongly upregulated by PBI and oleandrin, reaching a high level of statistical significance at the three highest concentrations (*p* < 0.01). Although PBI and oleandrin had milder effects on Poly I:C-challenged T cells (Figure 2C,D), the increased CD69 expression was highly significant (Figure 2C) compared to Poly I:C alone (*p* < 0.01). With both PBI and pure oleandrin, the expression levels of CD69 and CD25 on Poly I:C-challenged monocytes (Figure 2E,F) were upregulated, reaching statistical significance for the 31.3 ng/mL and the 125 ng/mL concentrations, respectively.

### 2.3. Direct Induction of Cytokine Production

**Figure 3:**
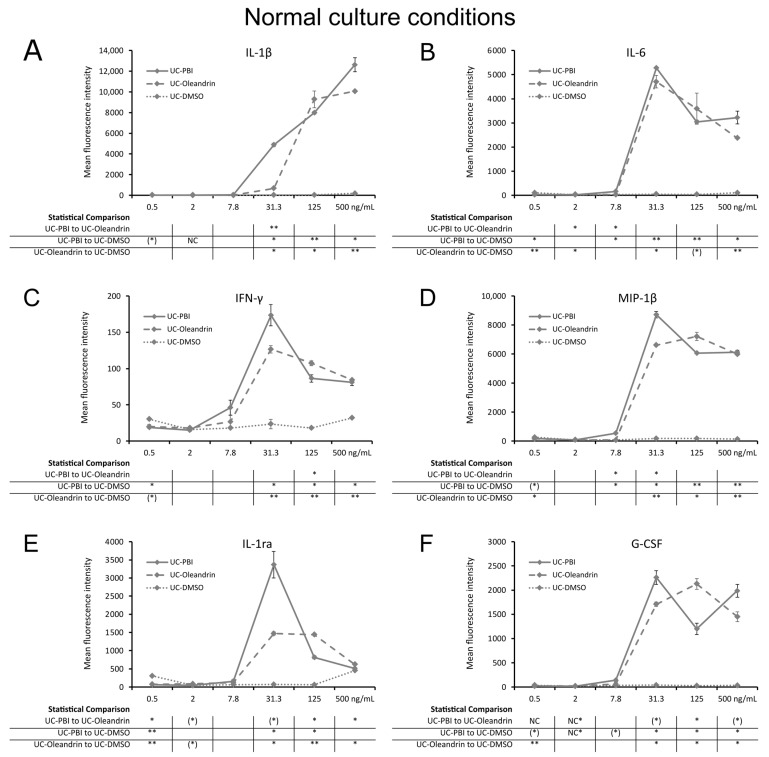
Cytokine levels in culture supernatants from 24 h PBMC cultures under normal culture conditions, where cells were treated with PBI (solid lines), oleandrin (dashed lines), and untreated control cultures treated with matching concentrations of DMSO (UC-DMSO) (dotted lines). (**A**) Interleukin-1 beta (IL-1β). (**B**) Interleukin-6 (IL-6). (**C**) Interferon-gamma (IFN-γ). (**D**) Macrophage inflammatory protein-1 beta (MIP-1β). (**E**) Interleukin-1 receptor antagonist (IL-1ra). (**F**) Granulocyte colony stimulating factor (G-CSF). Since both PBI and oleandrin were solubilized in DMSO, a matching concentration range of DMSO served as a solvent control. The results shown are representative of six repeats from different blood donors. For each concentration of a test product, results are shown as the average ± standard deviation of duplicate samples. In the table below the graph, statistical significance at different concentrations is indicated by asterisks, when *p* < 0.10: (*), *p* < 0.05: *, *p* < 0.01: ** and NC when not calculated.

Treatment of PBMC cultures with PBI and oleandrin had a direct effect on cytokine production (Figure 3). Evidence of direct immune activation by PBI and oleandrin was seen by the increased levels of four immune-activating pro-inflammatory cytokines including interleukin-1β (IL-1β; Figure 3A), interleukin-6 (IL-6; Figure 3B), interferon-gamma (IFN-γ; Figure 3C), and macrophage inflammatory protein-1beta (MIP-1β; Figure 3D), where the increased cytokine production was statistically significant for both test products at the three highest concentrations. In addition, the test products also induced the production of the anti-inflammatory cytokine, interleukin-1 receptor antagonist (IL-1ra; Figure 3E), with PBI inducing higher levels than oleandrin when administered at the 31.3 ng/mL concentration, reaching a statistical trend when compared to pure oleandrin (*p* < 0.1). More so, granulocyte colony stimulating factor (G-CSF; Figure 3F), a growth factor involved in stem cell mobilization, was also produced in cultures treated with PBI and oleandrin, reaching statistical significance for both products at the three highest concentrations (*p* < 0.05).

### 2.4. Regulation of Cytokines in Context of a Viral Mimetic Challenge

**Figure 4:**
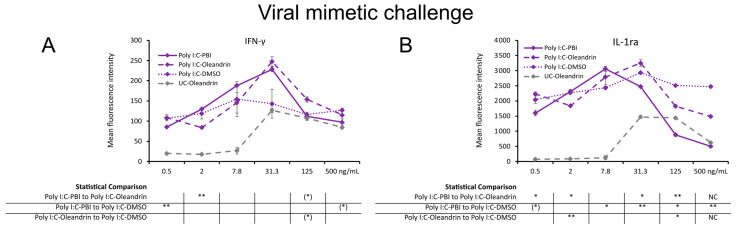
Cytokine levels in culture supernatants from 24 h PBMC cultures under a viral mimetic challenge, where the cells were treated with Poly I:C-PBI (solid lines), Poly I:C-oleandrin (dashed lines), and Poly I:C control cultures treated with matching concentrations of DMSO (Poly I:C-DMSO) (dotted lines), followed by a viral mimetic challenge by polyinosinic:polycytidylic acid (Poly I:C). (**A**) Interferon-gamma (IFN-γ). (**B**) Interleukin-1 receptor antagonist (IL-1ra). Since both PBI and oleandrin were solubilized in DMSO, a matching concentration range of DMSO served as a solvent control (Poly I:C-DMSO). The results shown are representative of six repeats from different blood donors. For each concentration of a test product, results are shown as the average ± standard deviation of duplicate samples. In the table below the graph, statistical significance at different concentrations is indicated by asterisks, when *p* < 0.10: (*), *p* < 0.05: *, *p* < 0.01: ** and NC when not calculated.

In PBMC cultures challenged with the viral mimetic polyinosinic:polycytidylic acid (Poly I:C), the production of IFN-γ (Figure 4A) was enhanced by PBI and oleandrin over Poly I:C alone and reached higher levels than in cultures treated with PBI and oleandrin without viral mimetic challenge. In contrast, the level of IL-1ra (Figure 4B) was down-regulated by PBI and oleandrin in cultures treated with PBI reaching a high level of statistical significance (*p* < 0.01). For oleandrin, the level of IL-1ra downregulation was milder, suggesting that non-oleandrin compounds in PBI could have contributed to the decrease in 1L-1ra expression.

### 2.5. Regulation of Inflammation

**Figure 5:**
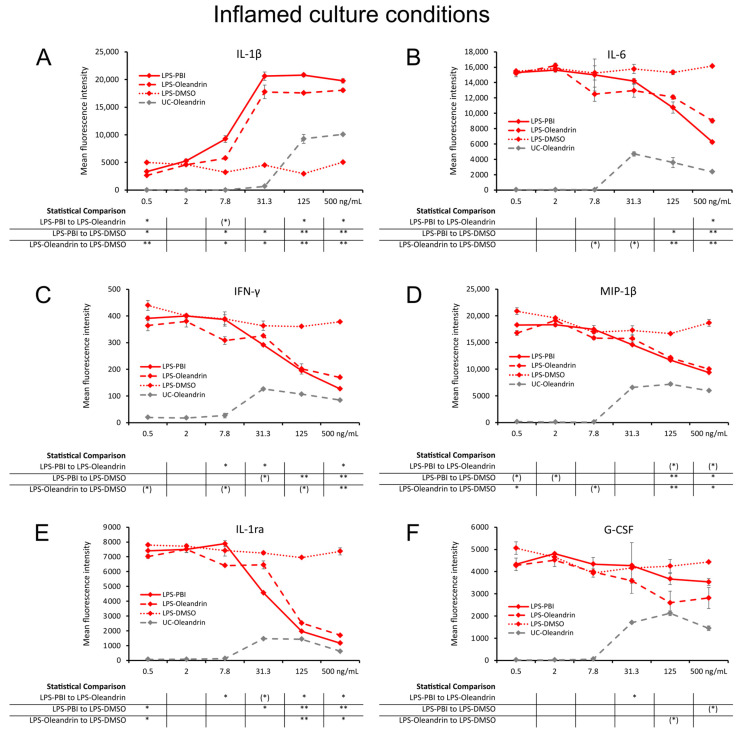
Cytokine levels in culture supernatants from 24 h PBMC cultures under inflamed culture conditions, where the cells were treated with LPS-PBI (solid lines), LPS-oleandrin (dashed lines), and LPS-treated control cultures treated with matching concentrations of DMSO (LPS-DMSO) (dotted lines), followed by an inflammatory challenge by lipopolysaccharides (LPS). (**A**) Interleukin-1 beta (IL-1β). (**B**) Interleukin-6 (IL-6). (**C**) Interferon-gamma (IFN-γ). (**D**) Macrophage inflammatory protein-1 beta (MIP-1β). (**E**) Interleukin-1 receptor antagonist (IL-1ra). (**F**) Granulocyte colony stimulating factor (G-CSF). Since both PBI and oleandrin were solubilized in DMSO, a matching concentration range of DMSO served as a solvent (LPS-DMSO). The results shown are representative of six repeats from different blood donors. For each concentration of a test product, results are shown as the average ± standard deviation of duplicate samples. In the table below the graph, statistical significance at different concentrations is indicated by asterisks, when *p* < 0.10: (*), *p* < 0.05: *, *p* < 0.01: **.

In cell cultures where LPS was added to induce inflammation, cultures that were pre-treated with either PBI or oleandrin showed evidence of complex modulation of inflammation (Figure 5). The test products enhanced LPS-induced IL-1β (Figure 5A) production, showing that the test products increased the LPS-induced macrophage activation. The increased levels of IL-1β were seen across a wide concentration range, with a high level of statistical significance at the highest two concentrations (*p* < 0.01) and statistical significance at 7.7 and 31.3 ng/mL concentrations (*p* < 0.05) compared to the control.

In contrast, the test products triggered reduced levels of LPS-induced IL-6 (Figure 5B), IFN-γ (Figure 5C), MIP-1β (Figure 5D), IL-1ra (Figure 5E), and G-CSF (Figure 5F), compared to the levels in control cultures treated with LPS alone. This inhibition of LPS-induced cytokine production by PBI and pure oleandrin was similar and reached a high level of statistical significance for either the 125 ng/mL or 500 ng/mL concentration for IL-6, IFN-γ, MIP-1β, and IL-1ra (Figure 5B–E). The inhibition of LPS-induced G-CSF (Figure 5F) production reached a statistical trend (*p* < 0.1).

### 2.6. Cytotoxic Activity

**Figure 6:**
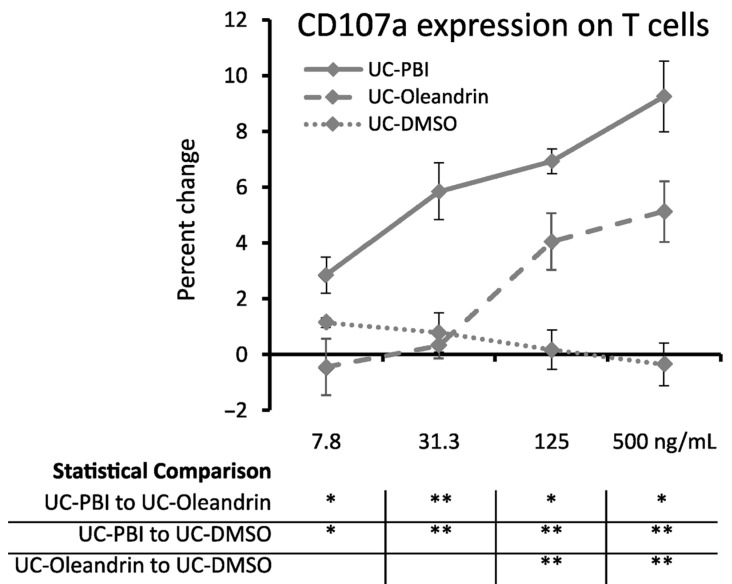
CD107a expression levels on CD3+ T cells from 4 h PBMC and K562 co-cultures treated with PBI (solid line), and oleandrin (dashed line), and DMSO (dotted line). Since both PBI and oleandrin were solubilized in DMSO, a matching concentration range of DMSO served as a solvent control (shown as a dotted line). The results shown are representative of three repeats on PBMC from different blood donors. For each concentration of a test product, results are shown as the average ± standard deviation of triplicate samples. In the table below the graph, statistical significance at different concentrations is indicated by asterisks, when *p* < 0.05: *, *p* < 0.01: **.

Peripheral blood mononuclear cells exposed to PBI and the pure compound oleandrin showed strongly enhanced cytotoxic killing activity against the MHC class-I deficient target cell line K562, using the gold-standard flow cytometric assay for CD107a expression. The increased expression of CD107a on PBI-treated T cells was highly significant (*p* < 0.01) when compared to matching concentrations of DMSO across the concentration range of 31.3–500 ng oleandrin/mL (Figure 6). The milder increase seen for oleandrin-treated T cells was only seen for the two highest concentrations of 125 and 500 ng oleandrin/mL, reaching a high level of statistical significance (*p* < 0.01). This shows that other components in the botanical PBI extract contribute to the effects on innate immune activation.

## 3. Discussion

This work was conducted to document the effects of the *Nerium oleander* botanical extract PBI 05204 (PBI) and one of its constituents, the glycoside oleandrin, on the innate immune system. From the results, there is strong evidence that both products directly activate specific types of immune cells that are essential to the innate immune system such as monocytes and natural killer (NK) cells. The mechanisms by which PBI and oleandrin activate resting immune cells are unknown. Although it has been shown that oleandrin modulates immune responses [27], the molecular mechanisms responsible for the highly selective activation of cells belonging to the innate immune system remain to be documented. 

The direct effects of PBI and oleandrin on innate immune cell activation were not detectable at 2 h. There were robust effects at both 8 and 24 h, where the 24 h results are reported here. There is evidence that oleandrin affects specific transcription factors [28], and most known effects on signal transduction pathways are inhibitory, including the effects on STAT2, MAPK, and NF-κB [19]. This timing suggests that a cascade of events is needed and takes place over the initial hours after exposure, leading to downstream immune cell activation. 

The expression levels of the two activation markers CD69 and CD25 on NK cells were higher in cell cultures treated with PBI than in cultures treated with oleandrin. The increased T cell cytotoxicity against the target cell K562 was stronger for PBI-treated cells than for oleandrin-treated cells. Both observations suggest that the effects of the botanical extract PBI are not entirely due to its content of oleandrin and that other compounds in the extract further contribute to the immune-activating effect.

Furthermore, PBI and oleandrin enhanced immune cell activation in response to a viral mimetic challenge, using synthetic double-stranded RNA to engage Toll-Like receptor-3 (TLR-3) on immune cells [29]. The expression of the activation markers, CD25 and CD69, on NK cells and monocytes was increased in cultures challenged with Poly I:C, when compared to cultures treated with test products only. Similarly, an increase in the production of IFN-γ was seen in cultures challenged with Poly I:C compared to test products-treated cultures only. This emphasizes a unique multi-faceted role for PBI and oleandrin in the treatment of viral illnesses by two separate and complementary mechanisms: (A) PBI and oleandrin are known to possess direct inhibitory properties against a broad spectrum of viruses affecting humans as well as commercial animal production, including HIV, Ebola, Marburg, HTLV-1, SARS-CoV-2, bovine viral diarrhea virus, bovine respiratory syncytial virus, and bovine coronavirus [16]; and (B) the products enhance immune cell activation in response to a viral challenge, including the activation of cells that play pivotal roles in destroying virally infected target cells [4]. Oleandrin and related extracts cause formation of progeny virions exhibiting significantly reduced infectivity, e.g. an 800-fold to 10,000-fold reduction in viral infectivity. In addition to its direct anti-viral effects, the selective elevated levels of IFN-γ suggest further enhancement of natural killer cell function [30]. The ability of a drug product to provide both direct (significant reduction in the infectivity of progeny virions) and indirect (balanced control of the innate immune response) antiviral activity is rare, if not unique.

In cultures exposed to an inflammatory challenge using the bacterial toxin LPS, the results showed that the two test products had selective immune-modulating effects, including a robust increase in IL-1β and RANTES levels, and a simultaneous decrease in other pro-inflammatory cytokines under these inflammatory conditions. We suggest that these complex data indicate that while the initial immune activation, leading to IL-1β production, is allowed to happen, PBI and oleandrin re-programmed the immune cells to exhibit less inflammatory responses, thus regulating and harnessing downstream inflammatory cascades. Further work is needed to understand the details of the signaling cascades involved, which cannot be explained by simple NF-κβ inhibition.

In situations where the immune system becomes overactive, causing ‘cytokine storms’, reduction in the LPS-induced inflammatory response is clinically relevant and suggests a beneficial role for PBI and oleandrin. It is important that PBI and oleandrin did not completely suppress the immune-activating pro-inflammatory cytokines but, instead, reduced them to similar levels of PBI or oleandrin in an inflammation-free environment. The concentration of LPS used in the cultures is highly inflammatory, and this nullification of LPS-induced elevation of production of IL-6, IFN-γ, MIP-1β, and IL-1ra cytokines by the highest concentrations of PBI and oleandrin points to a balanced control of inflammatory and anti-inflammatory response, which clinically could result in a robust innate immune response but with minimal tissue damage.

The clinical relevance of the data presented herein is potentially significant in terms of treatment or prevention of viral infections, because of the way oleandrin exerts its effect upon the innate immune system, particularly when considered in combination with its direct antiviral activity. Oleandrin promoted expression of cell-signaling cytokines, including IL-1b and IL-6, that alert other parts of the immune system that a foreign antigen (virus) is present. Oleandrin then stimulated activation of key innate immune cell types (monocytes/macrophages, NK cells) that are instrumental in fighting a viral infection. Oleandrin also induced expression of chemokines IFN-γ and MIP-1b, associated with increased ability to control active viral infections including HIV [31]. In a clinical situation, these cytokines and chemokines would also serve to recruit and activate cytotoxic CD8+ T lymphocytes and macrophages to initiate communication with cells of the adaptive immune system, leading to generation of immunological memory. Oleandrin mediated a proinflammatory immune response and facilitated a return to homeostasis by promoting the expression of anti-inflammatory cytokines such as IL-1ra.

The data suggest that the combined proinflammatory and anti-inflammatory activities, when translated to a clinical situation, support immune alertness and innate immune protection in combination with regulating effects that would protect from excessive inflammation. This may reduce tissue damage caused by extreme inflammation, as occurs during some viral infections. Finally, oleandrin promoted expression of restorative and rejuvenating growth factors, especially G-CSF, a growth factor involved in regenerative processes, stem cell mobilization, and supporting neurogenesis and neuroplasticity [32,33].

This in vitro work has a direct clinical relevance, since we hypothesize that similar events can occur when oleandrin or related extracts are administered in ways that introduce the products to mucosal membranes and activate the innate immune cells in the mucosa. Mucosal tissue is present in the respiratory, digestive, and reproductive systems. Consuming the products would introduce them to the gut mucosal immune system. In addition, nasal sprays containing PBI and oleandrin may provide protection of sinuses and airway mucosal membranes by interacting with immune cells residing in and selectively traffic through specific anatomical compartments [34,35,36]. In vivo animal data support this proposition, as evidenced by the prophylactic and therapeutic antiviral activities of oleandrin and PBI-06150, another *Nerium oleander* botanical extract, against SARS-CoV-2 infection, where it was demonstrated that PBI-06150 provided a statistically significant reduction in viral titer following direct administration of PBI-06150 to the nasal conchae (turbinate) of golden Syrian hamsters [37].

Topical application of PBI as well as related cardiac glycosides is of interest for the treatment of herpes outbreaks [22,38,39]. For example, another potential application is in wound healing [22,40], since pro-inflammatory immune activation is beneficial during the initial phase of healing, and the induction of the regenerative growth factor G-CSF may assist in the later phases of tissue recovery. Further research on the impact of oleandrin and related extracts on the kinetics and sequence of these events may provide clarification.

## 4. Materials and Methods

### 4.1. Reagents

Dulbecco’s phosphate-buffered saline (Gibco cat. # 141190-136), lipopolysaccharide (LPS) (Invitrogen cat. # 00-4976-93), Roswell Park Memorial Institute 1640 cell culture medium (Gibco cat. # 11835-030), Fetal Bovine Serum (Gibco cat. # A38401-01), penicillin–streptomycin 100× (Gibco cat. # 15140-122), CD69 fluorescein isothiocyanate (clone FN50, Invitrogen cat. # 11-0699-42), CD56 phycoerythrin (clone CMSSB Invitrogen cat. # 12-0567-42), and CD3 Super Bright 645 (clone OKT3 Invitrogen cat. # 64-0037-42) were purchased from Thermo Fisher Scientific (Waltham, MA, USA). CD25 Brilliant Violet 421 (clone 2A3 BD cat. # 564033), CD107a-FITC (clone H4A3 BD Pharmingen cat # 555800), and sodium heparin vacutainer tubes (BD cat. # 367878) were purchased from Becton-Dickinson (Franklin Lakes, NJ, USA). The human erythroleukemia cell line K562 was purchased from American Type Culture Collection (Manassas, VA, USA). Customized Bio-Plex Pro™ human cytokine arrays were purchased from Bio-Rad Laboratories Inc. (Hercules, CA, USA). Oleandrin (Sigma cat. # 06069-5MG) and Interleukin-2 (IL-2) (Sigma cat. # 17908-10KU) were purchased from Sigma-Aldrich Co. (St Louis, MO, USA). Lympholyte Poly (Cedarlane cat. # CL5070) was purchased from CedarLane (Burlington, NC, USA).

### 4.2. Test Products

PBI-05204 (PBI) is a supercritical CO2 extract of *Nerium oleander* leaves. It was provided by Phoenix Biotechnology, Inc. (San Antonio, TX, USA). Characterization of PBI-05204 was carried out using AccuTOF-DART mass spectrometer (Jeol UAS, Peabody, MA, USA). Specific content of the extract was previously reported [41]. The extract contains oleandrin (1.74% by weight) and compounds obtained from the plant material [42].

The two products were handled in the following manner: (1) A stock solution of PBI was prepared in DMSO, and subsequent dilutions were prepared in phosphate-buffered saline. The amount of PBI employed in the assays was normalized based upon its oleandrin content. (2) A stock solution of 5 mg/mL oleandrin was prepared in DMSO, and dilutions were prepared in phosphate-buffered saline. The range of concentrations in cell cultures was calculated based on the percentage of oleandrin and ranged from 0.5 to 500 ng oleandrin/mL cell culture medium. Matching concentrations of DMSO served as the solvent control.

### 4.3. Immune Cell Activation

Peripheral venous blood was drawn from healthy human donors upon written informed consent, as approval by the Sky Lakes Medical Center Institutional Review Board, Federalwide Assurance 2603. The blood was drawn into heparin vacutainer vials, and the peripheral blood mononuclear cells (PBMC) were isolated using Lympholyte Poly by centrifugation for 35 min at 400× *g*. The PBMC were washed twice in PBS and counted, and the density was adjusted to establish 0.18 mL cultures with a cell density at 106/mL, using Roswell Park Memorial Institute 1640 medium containing 10% fetal calf serum and 1% penicillin–streptomycin.

Serial dilutions of products or LPS (10 ng/mL) were added to cultures at a volume of 20 μL, so each culture well had a final volume of 0.2 mL/well. Cultures were then incubated at 37 °C, 5% CO_2_ for 24 h. The highly inflammatory LPS from *Escherichia coli* was used as a positive control for immune-cell activation. In parallel, IL-2 was used as a positive control for natural killer (NK)-cell activation, at a concentration of 100 IU/mL in cell culture. Untreated negative control cultures consisted of PBMC exposed to phosphate-buffered saline in the absence of test products. All treatments, including each concentration of test product and each positive and negative control, were tested in triplicate. After 24 h, blood cells were isolated from each culture well and stained for 15 min with fluorochrome-labeled antibodies at the recommended concentration. PBMCs were then fixed in formalin (%). The fluorescence intensities for CD3, CD25, CD56, and CD69 were measured by flow cytometry, using an Attune NxT acoustic-focusing flow cytometer (Thermo Fisher Scientific, Waltham, MA, USA). Data analysis utilized gating on forward scatter and side scatter to create electronic gates for the lymphocyte and monocyte/macrophage populations. The lymphocyte subpopulation was further analyzed for CD25 and CD69 expression on CD3-CD56+ NK cells, CD3+ CD56+ NKT cells, and CD3+CD56- T cells.

### 4.4. Production of Cytokines, Chemokines, and Growth Factors

After 2, 8, and 24 h of incubation, the supernatants were harvested from the PBMC cultures described above. Levels of 27 cytokines and chemokines were quantified using Bio-Plex protein arrays (Bio-Rad Laboratories Inc., Hercules, CA, USA) and utilizing xMAP technology (Luminex, Austin, TX, USA). The cytokine array included: IL-1β, IL-1ra, IL-2, IL-4, IL-5, IL-6, IL-7, IL-8, IL-9, IL-10, IL-12 (p70), IL-13, IL-15, IL-17, Eotaxin, basic FGF, G-CSF, GM-CSF, IFN-γ, IP-10, MCP-1 (MCAF), MIP-1α, MIP-1β, PDGF-BB, RANTES, TNF-α, and VEGF.

### 4.5. NK Cell Cytotoxicity towards K562 Human Myelogenous Leukemia Target Cells

The cell surface expression of CD107a (LAMP-1) is a marker for secretory degranulation of NK and activated cytotoxic T cells when they are co-cultured with malignant or virus-infected target cells [43]. Peripheral blood mononuclear cells from healthy human donors were co-cultured with K562 cells in the absence versus presence of test products or matching concentrations of the DMSO solvent control. All treatments, including each concentration of test product and each positive and negative control, were tested in triplicate. CD107a-FITC antibody (6.5 µL/well) was added at the beginning of each culture, along with the protein transport inhibitor BD GolgiStop™, which contains Monensin and blocks intracellular protein transport processes. After 4 h of incubation at 37 °C, 5% CO_2_, the cells were washed in phosphate-buffered saline and stained with CD3-PerCP and CD56-PE antibodies and then fixed in formalin. Flow cytometry analysis was performed where the strategy involved initial gating of the lymphocyte and monocyte subpopulations using the forward/side scatter plot, followed by analysis of T cells and NK cells in the lymphocyte population. The fluorescence intensity of CD107a was evaluated as the mean fluorescence intensity on monocytes, NK cells, and T cells. 

### 4.6. Statistical Analysis

Average and standard deviation for each data set was calculated using Microsoft Excel. Statistical analysis of data was performed using the 2-tailed, independent *t*-test. Statistical significance was set at *p* < 0.05, with a higher level of significance *p* < 0.01.

## 5. Conclusions

The results presented here show that PBI and oleandrin have direct immune-activating activities that promote a controlled and balanced innate immune response. They directly activate innate immune cells, enhance anti-viral immune responses through NK cell activation and IFN-γ levels, and modulate immune responses under inflamed conditions. 

Further work should focus on signaling cascades in normal versus inflamed culture conditions and the role of monocytes in initiating these events, as well as monocyte M1 versus M2 polarization. Evaluation of T cell subsets is also necessary including γδT cells, CD8+ cytotoxic T cells, and regulating T cells. These data suggest that topical application to control herpes outbreaks and the use of inhalable, intranasal, or oral formulations for the treatment of upper respiratory tract infections deserve appropriate clinical evaluations.

Finally, it is worth reemphasizing the rarity of a single molecule, such as oleandrin, and extracts containing it, to be able to provide such a robust multifaceted and beneficial effect upon the innate immune system, particularly at nanomolar concentrations. The direct effects upon the innate immune system in combination with the direct antiviral effects, which also occur at nanomolar concentrations, have the potential for significant clinical impact and warrant their clinical evaluation, particularly in the treatment of viral infections that directly or indirectly induce a hyperinflammatory immune response.

## Data Availability

The data presented in this study are available on request from the corresponding author upon reasonable request.
